# 

**Published:** 1890-11-01

**Authors:** 


PRICE ONE PENNY.

No. 214, Vol. IX.—November 1st, 1890.

[Registered at the General Post Office as a Newspaper for transmission in the United Kingdom and Abroad.]

WITH EXTRA NURSING SUPPLEMENT.

Per Annum, 6s. 6d.; post free.

Monthly Parts, 6d.; post free 8d.

Ditto per Annum, 8s., post free.

### THE HOSPITAL

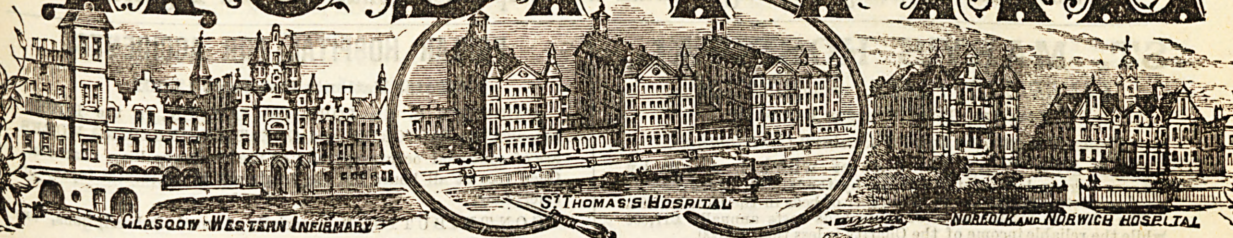

A WEEKLY JOURNAL OF

Science, Medicine, Nursing and Philanthropy.

### SATURDAY, NOVEMBER 1st, 1890.

**What Says the Student?** . . . . . . . 65
**Passing Topics.**—Hospital Choirs . . . . . . 66
**Physical Education in America** . . . . . . 67
**Working Women in Large Towns.**—XIV.—Some Sources of Distress . . . . . . . . . . 68
**The Doctor's Armchair.**—The Life Problem at Forty . 69
**The History and Capabilities of Herbal Simples**—By W. T. Fernie, M.D.—XXII.—Coltsfoot . . . . 70
**Everybody's Page** . . . . . . . . . 71
**Notes and News** . . . . . . . . . 72
**The Story of the Insane from Year to Year** . . . 73
**Annotations.**—London's Lunatics and Provision for Them—Science Broadens Slowly Down . . . . . . 74
**The Practitioner's Mirror and Retrospect:**—
MEDICINE.—Poisoning by Colchicin—Treatment of Detached Retina by Iodine Injections into the Bulb . . . . 75
THE LONDON POST-GRADUATE COURSE AT THE PADDINGTON INFIRMARY—Diseases of the Bladder and Prostate, &c. . 75
THE MEDICAL SCHOOLS—Entries, Winter Session, 1890-91 . 77
NEW DRUGS, APPLIANCES, AND THINGS MEDICAL . . 77
**The Greatest Medical Library of the World** . . 78
**General Charities—Scraps and Gleanings** . . . 79
**The Student of Medicine:**—Editorial—Notes from the Schools—Men of the Future—Students' Medical Societies, 80; Scholarships and Prizes, Third List—Pass List—Athletics, 83; Events for November . . . . . . 84

### EXTRA SUPPLEMENT—THE NURSING MIRROR.

**En Passant.**—To Our Readers—The Fall—Tynemouth Infirmary—Nurses at Norwich—So Irish, You Know! . . xvii

**Lectures on Surgical Ward Work and Nursing.** By Alex. Miles, M.B.Edin., C.M., F.R.C.S.E.—Introduction—Lecture I. Antiseptics . . . . . . . xviii

**Six Months in a Hospital.** By a "Special Pro."—V. . xix

**Words of Consolation.**—The Great Physician . . . xix

**Nurses on Their Travels.**—Private Nursing in Paris . xx

**Death in Our Ranks—Ward Tattle** . . . . . . xx

**Everybody's Opinion.**—The Brassey Home—Male Nurses—A Disclaimer . . . . . . . . . . xxi

**Presentation** . . . . . . . . . . xxi

**Notes and Queries** . . . . . . . . . xxi

**Off Duty.**—The Hare-lipped Baby . . . . . . xxii

**Lectures and Amusements** . . . . . . . xxii

**Amusements and Relaxation** . . . . . . . xxii

